# Molecular actions of two synthetic brassinosteroids, iso-carbaBL and 6-deoxoBL, which cause altered physiological activities between Arabidopsis and rice

**DOI:** 10.1371/journal.pone.0174015

**Published:** 2017-04-03

**Authors:** Ayako Nakamura, Naoya Tochio, Shozo Fujioka, Shinsaku Ito, Takanori Kigawa, Yukihisa Shimada, Makoto Matsuoka, Shigeo Yoshida, Toshinori Kinoshita, Tadao Asami, Hideharu Seto, Takeshi Nakano

**Affiliations:** 1 RIKEN, Wako, Saitama, Japan; 2 RIKEN Systems and Structural Biology Center, Tsurumi, Yokohama, Kanagawa, Japan; 3 Graduate School of Agricultural and Life Science, The University of Tokyo, Yayoi, Bunkyo, Tokyo, Japan; 4 RIKEN, Plant Science Center, Tsurumi, Yokohama, Kanagawa, Japan; 5 Bioscience and Biotechnology Center, Nagoya University, Chikusa, Nagoya, Aichi, Japan; 6 Division of Biological Science, Graduate School of Science, Nagoya University, Chikusa, Nagoya, Japan; 7 JST-CREST, Kawaguchi, Saitama, Japan; NARO Institute of Agrobiologial Sciences, JAPAN

## Abstract

Brassinosteroid (BR) is an important plant hormone that is perceived by the BRASSINOSTEROID INSENSITIVE 1 (BRI1) receptor. BRI1 is conserved among dicot and monocot species; however, the molecular mechanism underlying BR perception in monocots is not fully understood. We synthesised two BRs, iso-carbabrassinolide (iso-carbaBL) and 6-deoxoBL, which have different BR activities in *Arabidopsis thaliana* (Arabidopsis) and rice. Our bioassay indicated that iso-carbaBL has relatively strong BR activity in Arabidopsis, but is inactive in rice and competitively inhibits BR activity. The bioactivity of 6-deoxoBL was similar to that of BL in Arabidopsis, but was much lower in rice. Binding experiments using recombinant Arabidopsis and rice BRI1 protein fragments suggested that iso-carbaBL and 6-deoxoBL bind to both receptors. These results showed that iso-carbaBL and 6-deoxoBL act as an antagonist and agonist, respectively, of BRs in rice. A docking simulation analysis suggested that iso-carbaBL fits deeper in the binding pocket to block the binding of active BR to rice BRI1. The simulated binding energy of 6-deoxoBL with rice BRI1 is much lower than that with Arabidopsis BRI1. The possible structural characteristics of rice BRI1 were determined based on the difference in the BR activities of iso-carbaBL and 6-deoxoBL in Arabidopsis and rice.

## Introduction

Brassinosteroids (BRs) are the only steroidal hormones in plants and have unique biological effects on plant growth and development. The first BR to be isolated was brassinolide (BL), the most active natural BR. The structure of BL was determined by Grove and colleagues as (*22R*,*23R*,*24S*)-2α,3α,22,23-tetrahydroxy-24-methyl-B-homo7-oxa-5α-cholestan-6-one[1]. The second active natural BR, castasterone (CS), the precursor of BL, was isolated and determined as (*22R*,*23R*,*24S*)-2α,3α,22,23-tetrahydroxy-24-methyl- 5α-cholestan-6-one [2]. BL has been isolated from various dicot plants, and although bioassays such as lamina joint inclination assays show that BL has strong BR activity in rice, BL has not been isolated from rice or other monocot plants, and several studies suggest that CS is the end BR product in rice [3–6].

The BR receptor, BRASSINOSTEROID-INSENSITIVE 1 (BRI1), was identified from an Arabidopsis brassinosteroid-insensitive mutant, *bri1* [7,8]. The Arabidopsis BRI1 protein is a serine/threonine kinase with an extracellular domain containing 25 leucine-rich-repeats (LRRs), which are interrupted by a 70-residue island domain (ID) at the 21^st^ LRR [8]. The homologous gene for Arabidopsis BRI1 in rice shares the same domain organisation as Arabidopsis BRI1, except that the number of the LRRs is 22 [9]. The activity of Arabidopsis BRI1 as a BR receptor was demonstrated using [^3^H]-BL in 2001 [10]. Using biotin-tagged photoaffinity CS (BPCS), the importance of the ID and the 22^nd^ LRR of Arabidopsis BRI1 for BR binding was determined [11]. The authors also showed that the first five amino acid residues of the ID and the last eight amino acid residues of the 22^nd^ LRR are critical for BR binding. Recently, the crystal structure of BRI1 was determined using the Arabidopsis BRI1 ectodomain [12,13]. BRI1 exists as a monomer in crystals [13]. The ID folds back into the interior of the superhelix to create a surface pocket for BL binding. It was also suggested that binding of the hormone to BRI1 generates a docking platform for a co-receptor that is required for receptor activation [12]. Structural studies of BRI1 and BL were performed based on demonstration of the BL-dependent interaction of BRI1 and SERK1 or BAK1 (SERK3) ectodomains, the co-receptors of BRI1 [14,15]. Based on these results, the role of BL as molecular glue in BRI1 and SERK1 binding and the contribution of 2α and 3α hydroxyls in BL to the interaction with SERKs were demonstrated. BR-mediated heterodimerization of BRI1 and BAK1 induces sequential transphosphorylation of the complex and subsequently enhances BR signaling [16].

**Fig 1:**
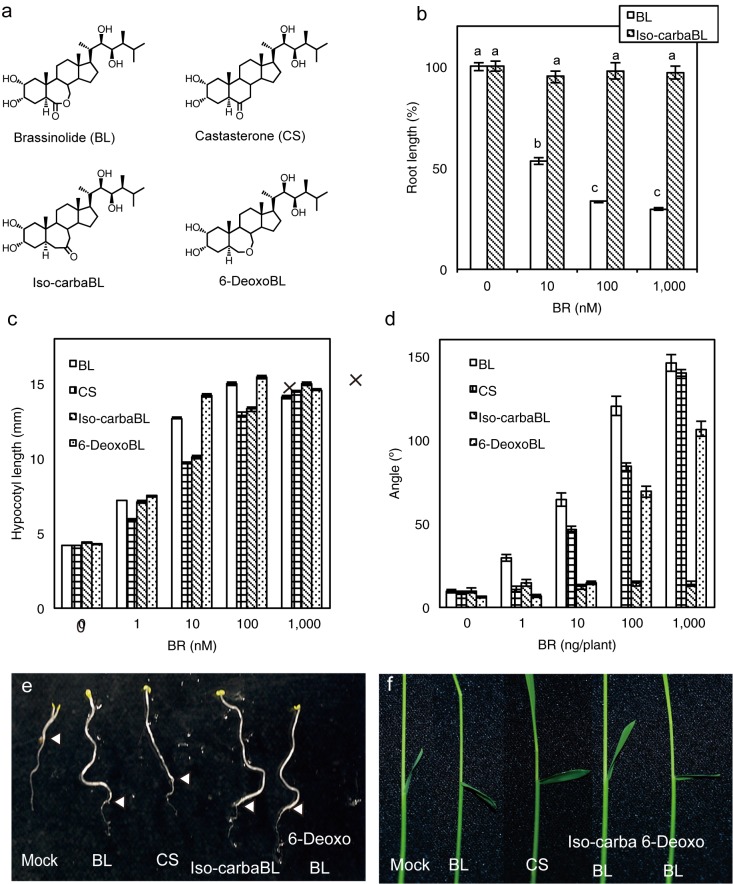
BR activities of iso-carbaBL and 6-deoxoBL. (a) Chemical structures of brassinolide (BL), castasterone (CS), iso-carbaBL, and 6-deoxoBL. (b) Wild-type (WT) plants were grown in an MS agar medium containing 3% sucrose at the indicated concentrations of BL or iso-carbaBL for 7 d, after which root length was measured. Data are represented as means ± SE (n > 14, **p < 0.01, Tukey-Kramer test. The difference was indicated by alphabetical order) (c) BR activities of BL, CS, 6-deoxoBL, and iso-carbaBL in Arabidopsis were analysed. *det2-1* plants were grown for 8 d in the dark, after which hypocotyl length was measured. Data are represented as means ± SE (n = 15). (d) BR activities of BL, CS, iso-carbaBL and 6-deoxoBL in rice were analysed. WT plants were grown for 4 d under continuous light and treated with BRs for another 2 d, after which the angle of the lamina joint was measured. Data are presented as means ± SE (n = 10). (e) Response to 1 μM BRs in Arabidopsis. The white triangles indicate the border of hypocotyl and root. (f) Lamina joint angle in response to 100 ng BRs. The experiments were repeated twice with similar results.

Previously, we found that the synthesised BR analogues, 6a-carbaBL and 6-Deoxo-6a-oxo-6a-carbaBL (iso-carbaBL; Fig 1A), exhibited different BR activities than BL in Arabidopsis based on *det2* hypocotyl elongation (DHE) assays and in rice based on rice lamina inclination (RLI) assays [17]. These analyses supported research strategies using structure-activity relationships with BR analogues; however, biological analyses of these compounds have not been performed. To explore the detailed mechanism of BR analogues, we performed a detailed analysis of iso-carbaBL and another synthesized BL, 6-deoxobrassinolide (6-deoxoBL, Fig 1A), based on binding analysis to Arabidopsis and rice BRI1 and expression analysis of BR-response marker genes.

## Results

### BR activity of iso-carbaBL and 6-deoxoBL in Arabidopsis and rice

We applied the DHE assay using Arabidopsis *det2*, a BR deficient mutant, and the RLI assay using *Oryza sativa* L. cv. Nipponbare to evaluate the BR activities of iso-carbaBL and 6-deoxoBL in Arabidopsis and rice [17,18] (Fig 1). The DHE and RLI assays results were compared with those obtained using BL and CS. Iso-carbaBL rescued the dwarf hypocotyl phenotype of *det2* and its activity was relatively stronger than that of CS in Arabidopsis (Fig 1C and 1E); however, it did not promote inclination of the lamina joint and did not show BR activity in rice (Fig 1D and 1F). To investigate the tissue specificity of iso-carbaBL activity, we also analysed the effect of iso-carbaBL on root growth in rice. Root growth was inhibited by a high concentration of BL, but was not affected by iso-carbaBL treatment (Fig 1B). This result strongly suggested that iso-carbaBL is inactive in the whole rice plant body. In Arabidopsis, 6-deoxoBL strongly rescued the dwarf hypocotyl phenotype of *det2* and the activity was comparable to that of BL, the most active natural BR (Fig 1C and 1E). In rice, 6-deoxoBL promotes lamina inclination; however, its effect was relatively weaker than that of CS and the activity was approximately 10-fold weaker than that of BL (Fig 1D and 1F).

**Fig 2:**
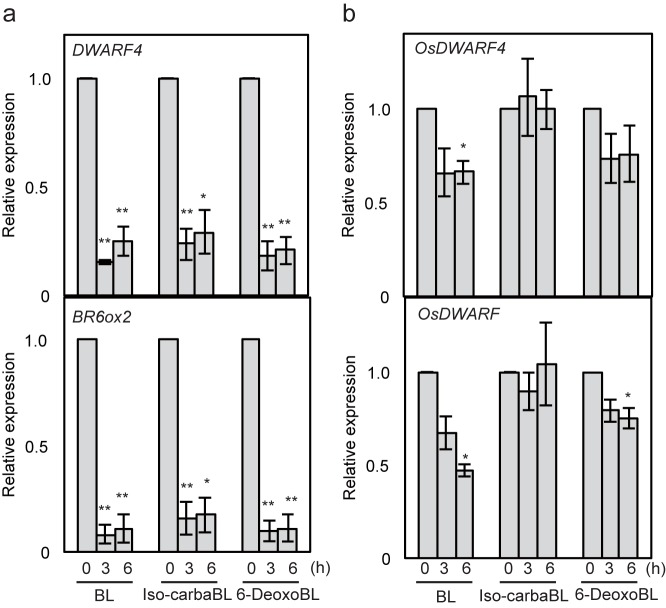
Feedback responses of BR biosynthetic gene expression to BRs in Arabidopsis and rice. Seven-day-old Arabidopsis and rice plants were treated with 1 μM BL, 6-deoxoBL, or iso-carbaBL for the indicated number of hours and the transcript levels of the *DWARF4* and *BR6ox2* genes of Arabidopsis (a) and the *OsDWARF4* and *OsDWARF* genes of rice (b) were analysed. Data are representative of three independent experiments (n = 3, error bars are ± SE, *p < 0.05, **p < 0.01, Student’s *t-*test against the data for each 0 h).

To further confirm the BR activity of iso-carbaBL and 6-deoxoBL, we used quantitative reverse transcription-polymerase chain reaction (qRT-PCR) analysis to investigate BR-biosynthetic gene expression, which is decreased in a feedback manner by active BRs in Arabidopsis and rice. In Arabidopsis, the expression levels of two BR biosynthetic genes, *DWARF4* and *BR6ox2*, C22-hydroxylase and C6-oxidase, respectively, were decreased significantly within 3 h in response to BL treatment (Fig 2A). In Arabidopsis, iso-carbaBL and 6-deoxoBL treatments also decreased BR biosynthesis gene expression to levels similar to those with BL (Fig 2A). In rice, the expression levels of *OsDWARF4* and *OsDWARF*, the rice homologs of the Arabidopsis *DWARF4* and *BR6ox2* genes, respectively, were decreased gradually within 6 h by BL or 6-deoxoBL treatment, but not by iso-carbaBL treatment (Fig 2B). The response to BRs in rice was modest as compared to that of Arabidopsis, but the tendency was similar to that in public database (RiceXPro, http://ricexpro.dna.affrc.go.jp). These results were similar to the biological activities of iso-carbaBL and 6-deoxoBL shown in Fig 1, both in Arabidopsis and rice.

**Fig 3:**
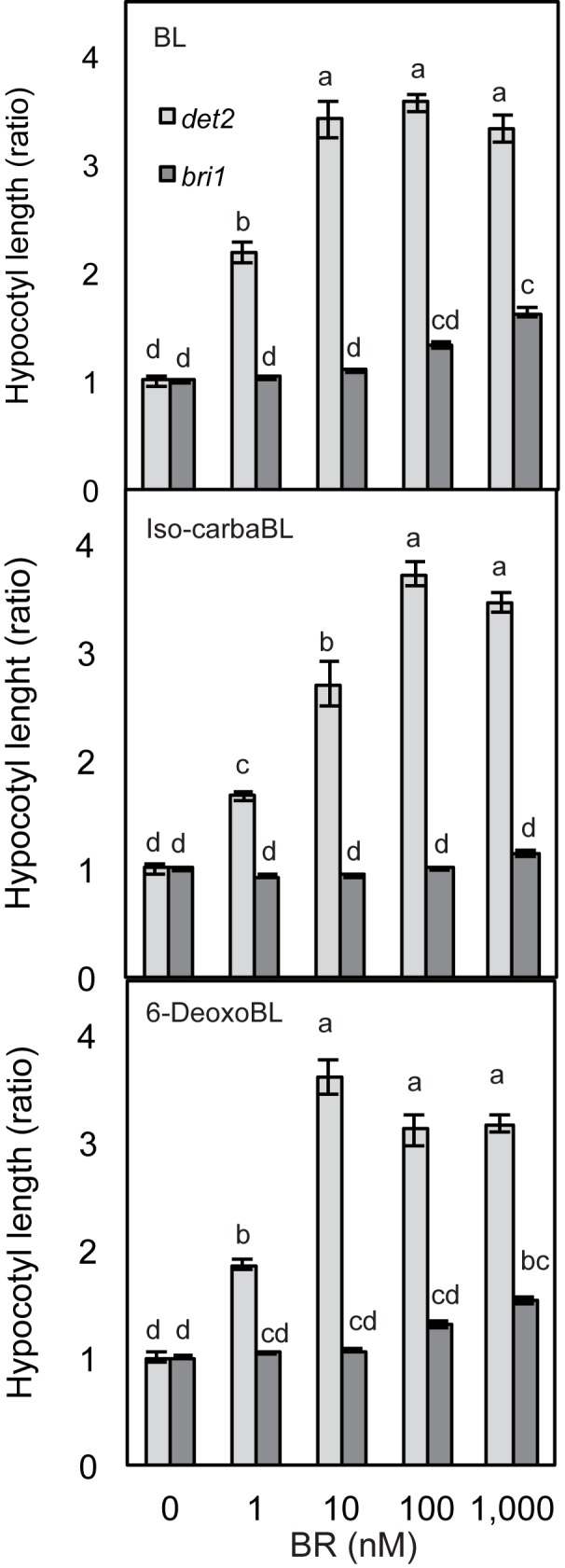
Response to 6-deoxoBL and iso-carbaBL in *bri1-5*. *det2-1* and *bri1-5* seedlings were treated with BL (top), iso-carbaBL (middle), or 6-deoxoBL (bottom) for 8 d in the dark, after which hypocotyl length was measured. Data are means ± SE (n = 15, *p* < 0.01 Tukey-Kramer test The difference was indicated by alphabetical order).

We also confirmed that the BR activities of iso-carbaBL and 6-deoxoBL are mediated by BRI1, the main BR receptor, in Arabidopsis. We performed hypocotyl elongation assays using a BRI1-deficient mutant, *bri1-5*, which showed weak BR receptor activity [19]. The hypocotyl elongation stimulated by iso-carbaBL was almost completely suppressed in *bri1-5* (Fig 3, middle). At high concentrations, BL and 6-deoxoBL induced hypocotyl elongation, even in *bri1-5* (Fig 3, top and bottom).

#### Binding of iso-carbaBL and 6-deoxoBL to Arabidopsis and rice BRI1 fragments

**Fig 4:**
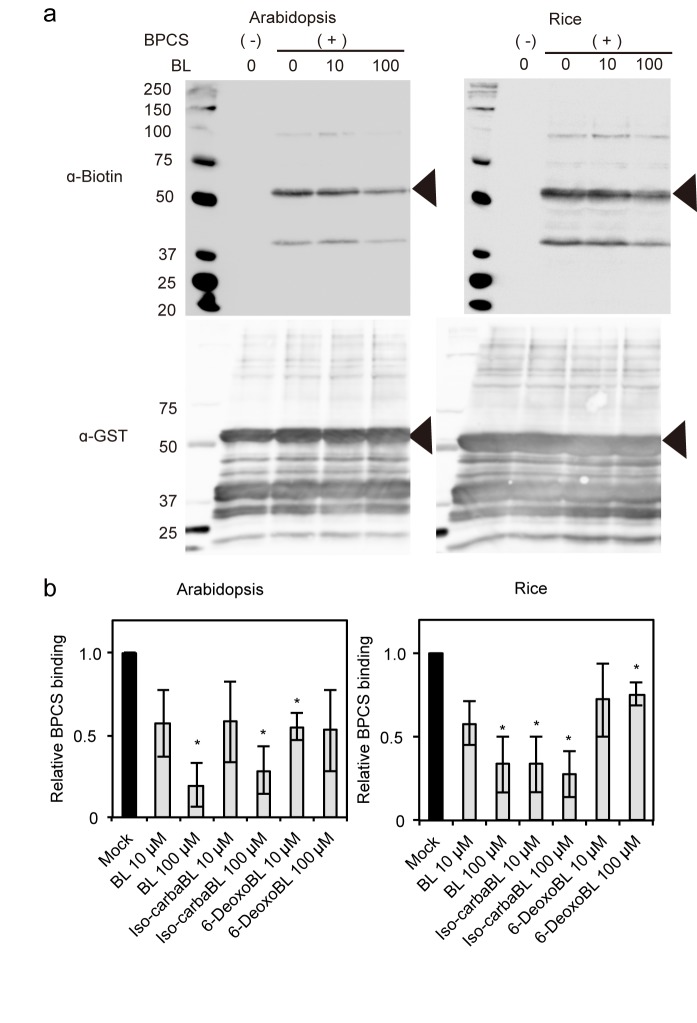
Binding of BRs to Arabidopsis and rice BRI1 recombinant proteins. (a) Representative full-length gel blot image of competitive inhibition of BPCS binding to Arabidopsis LRR21-ID-LRR22 or rice BRI1 LRR17-ID-LRR18 by BL. (b) Competitive inhibition of BPCS binding to Arabidopsis LRR21-ID-LRR22 or rice BRI1 LRR17-ID-LRR18 by BL, iso-carbaBL and 6-deoxoBL. Images were quantified using ImageJ64 (1.48v) software. Biotin expression was normalized to GST expression and the value relative to the mock treatment is indicated. Data are means ± SE (n = 3, *p < 0.05, Student’s *t*-test against the data for mock treatment).

To clarify the detailed molecular functions of iso-carbaBL and 6-deoxoBL as BL analogs, we examined their binding activities to BRI1 in Arabidopsis and rice. Previously, Kinoshita et al. demonstrated that the 70-amino acid ID located between the 21^st^ and 22^nd^ LRRs of Arabidopsis BRI1 and the 22^nd^ LRR were essential domains for BR binding [11]. The rice BRI1 encodes 22 LRRs and an ID between the 17^th^ and 18^th^ LRRs in the extracellular domain [9]. To examine the binding of iso-carbaBL and 6-deoxoBL to BRI1, we produced Arabidopsis and rice BRI1 recombinant protein fragments containing the ID and the neighboring LRRs corresponding to the core region of BR binding determined in Arabidopsis BRI1 [11]. As demonstrated using the Arabidopsis BRI1 fragment, biotin-tagged photoaffinity CS (BPCS) also bound to the rice BRI1 core region (Fig 4A, lane BPCS(+)BR (0 μM).), which demonstrated that the ID and the neighboring LRRs (LRR17-ID-LRR18) of rice BRI1 are sufficient for BR binding. We then determined the binding abilities of iso-carbaBL and 6-deoxoBL to BRI1 fragments based on competition assays. BL, the positive control, inhibited the binding of BPCS to both Arabidopsis and rice BRI1 in a dose-dependent manner (Fig 4A). Iso-carbaBL, which is inactive in rice, decreased the binding of BPCS not only to Arabidopsis BRI1, but also to rice BRI1 in a dose-dependent manner (Fig 4B). Although it did not show clear dose-dependency, 10 μM or 100 μM 6-deoxoBL decreased the binding of BPCS to Arabidopsis or rice BRI1 protein fragments, respectively (Fig 4A and 4B).

#### Mode of action of iso-carbaBL in Arabidopsis and rice

**Fig 5:**
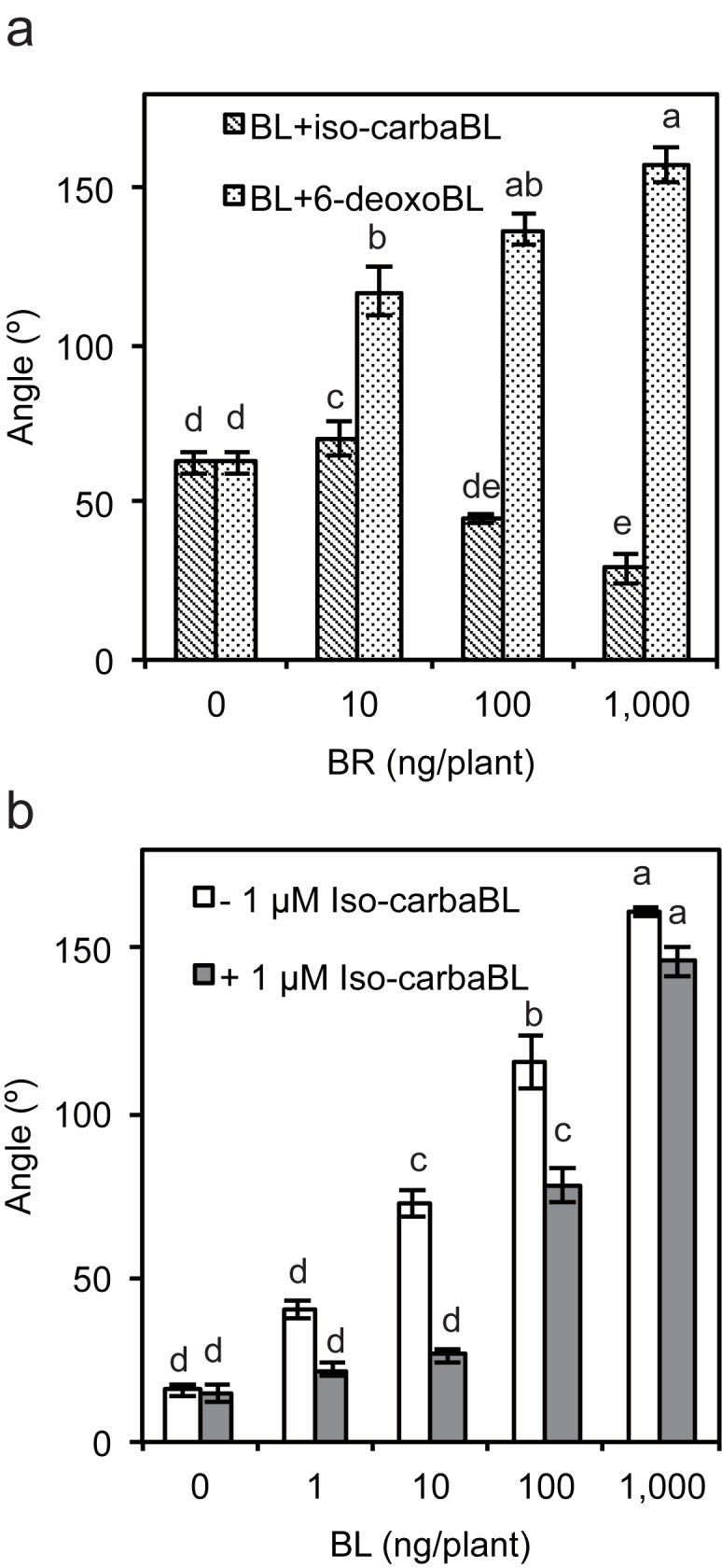
Effects of 6-deoxoBL and iso-carbaBL on the BL-induced lamina joint response in rice. (a) Four-day-old WT rice plants were treated with 6-deoxoBL or iso-carbaBL with or without 10 ng BL for 2 d and the resultant bending angle of the lamina joint was measured. (b) Four-day-old WT rice plants were mock treated (0.01% DMSO) or treated with 1 μg of iso-carbaBL for 1 h and then treated with 0, 1, 10, 100, or 1,000 ng of BL for 2 d, after which the resultant bending angle of the lamina joint was measured. Data are means ± SE (n ≥ 8, *p* < 0.01, Tukey-Kramer test The difference was indicated by alphabetical order)). Three biological replicates of each sample type were tested.

Since iso-carbaBL bound to rice recombinant protein and showed no BR activity, we hypothesized that this chemical functions as an antagonist of active BRs in rice. To explore this possibility, we performed an RLI test to investigate the response to BL in combination with iso-carbaBL or 6-deoxoBL in rice. As the amount of co-treated iso-carbaBL increased, the bending angle of the lamina joint induced by BL was abolished (Fig 5A). The combination of 6-deoxoBL and BL enhanced the bending of lamina joint additively. Thus, iso-carbaBL and 6-deoxoBL function differently in rice (Fig 5A). To further confirm the antagonistic effect of iso-carbaBL, we conducted a second RLI test. Rice lamina joints were treated with or without 1 μg of iso-carbaBL and then treated with various concentrations of BL, and the resultant lamina joint angles were measured. The lamina joints responded to 1 ng of BL in the absence of iso-carbaBL, but required more than 10 ng of BL when treated with iso-carbaBL (Fig 5B).

### Modelling of Arabidopsis and rice BRI1 binding with BL, iso-carbaBL, and 6-deoxoBL

To compare the modes by which BL, iso-carbaBL, and 6-deoxoBL bind Arabidopsis and rice BRI1, we performed protein-ligand docking simulation analyses. The protein structure of rice BRI1 was modeled using the MODELLER programme [20] (version 9v3) based on the crystal structure of the extracellular domain of Arabidopsis BRI1 [13] (3RGZ). We then performed an AMBER simulation [21–23] (version 9) for all complexes with explicit water molecules until we obtained well-converged structures. Binding enthalpy changes were evaluated based on the molecular mechanics Poisson-Boltzmann surface area (MM-PBSA) method implemented in the AMBER programme and the number of hydrogen bonds and van der Waals contacts between BRs and BRI1s were estimated using the LIGPLOT programme [24] (version 4.5.3). The binding enthalpy of the modeled rice BRI1 ligand-binding domain to BL was similar to that of Arabidopsis BRI1.

**Fig 6:**
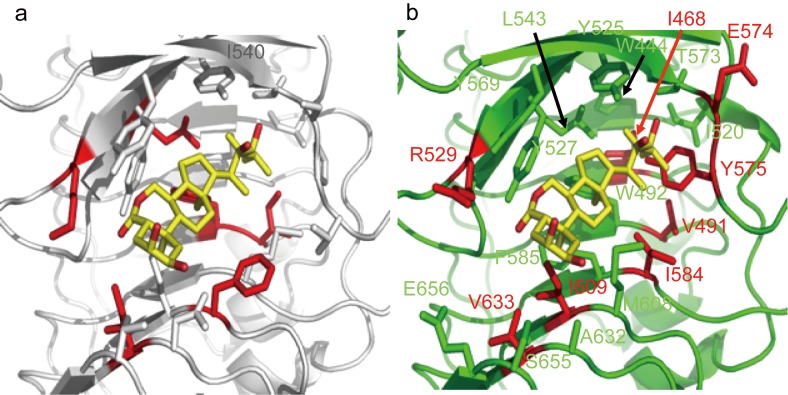
Structural model of the core region for BR binding in Arabidopsis and rice BRI1. **A,** The crystal structure of the Arabidopsis binding domain (PBD ID: 3RGZ) [13]. B, The calculated protein-ligand interaction of rice BRI1 and BL. The structure was calculated based on the coordinates of 3rgz and the amino acid sequence of the external domain of rice BRI1. The position of BL does not reflect the results of estimation.

The simulated binding enthalpy changes suggested that iso-carbaBL and 6-deoxoBL binding to Arabidopsis BRI1 was relatively weaker than that of BL, possibly because of the lack of a hydrogen bond between BRs and K601 (Table 1). This is plausible because iso-carbaBL and 6-deoxoBL lack the carbonyl group at the C6 position, which forms a hydrogen bond with K601 in Arabidopsis BRI1 (Figs 1 and 6). The results of MM-PBSA analyses suggested that BL could bind to rice BRI1 with a binding enthalpy similar to that of Arabidopsis BRI1. In contrast, the binding enthalpies of iso-carbaBL and 6-deoxoBL to rice BRI1 (–29.19 and –29.15 kcal/mol, respectively) showed significantly weaker bindings than that of BL (–39.06 kcal/mol) (Table 1). These values were also much higher which mean lower bindings, than the binding enthalpies of iso-carbaBL and 6-deoxoBL to Arabidopsis BRI1 (–35.90 and –37.26 kcal/mol, respectively). Docking simulation analyses suggested that lack of hydrogen bonding between BRs and R529 in rice BRI1 decreased the binding enthalpy, as observed for Arabidopsis BRI1 (Fig 6 and Table 1). In addition, we could not identify any hydrogen bonds between E574 in rice BRI1 and BRs among the BR-complex structure ensembles. Considering that the corresponding hydrogen bonds between S647 and BRs were maintained in the Arabidopsis BRI1 and BRs complex, the breakage of the hydrogen bond in rice BRI1 was caused by the shift in position of BRs, which might be induced by the enhanced flexibility of Y575 corresponding to P648 in Arabidopsis BRI1 (Fig 6). In the BL and rice BRI1 complex, the degree of this conformational shift was small to form the corresponding hydrogen bond by the other hydrogen bond between another side (carbonyl group at the C6 position) of BL and R529 (Fig 6B and Table 1). However, in the BRs and rice BRI1 complex, the lack of the carbonyl group at the C6 position and the flexibility of Y575 relative to P648 in Arabidopsis BRI1 did not allow for formation of hydrogen bonds found in the BL-BRI1 complex (Figs 1 and 6). In addition, since iso-carbaBL has another carbonyl group at the C7 position (Fig 1), this carbonyl group may keep a distance from R529 in rice BRI1 so that the iso-carbaBL can move further inside BRI1 into the space induced by the flexibility of Y575 of rice BRI1, consequently, making contacts with V491 (Fig 6 and Table 1). In contrast, since 6-deoxoBL does not have this carbonyl group at the 7 position, 6-deoxoBL can contact R529 despite the lack of the hydrogen bond provided by the carbonyl group at the C6 position to maintain the structural position in the complex with rice BRI1 without any contacts with V491 (Fig 6 and Table 1).

**Table 1:** Protein-ligand interactions of AtBRI1 and OsBRI1 and BL, iso-carbaBL and 6-deoxoBL.

AtBRI1	BL		Iso-carbaBL		6-DeoxoBL		OsBRI1	BL		Iso-carbaBL		6-DeoxoBL	
res.	h-bond	VdW	h-bond	VdW	h-bond	VdW	res.	h-bond	VdW	h-bond	VdW	h-bond	VdW
W516	—	—	—	—	—	—	W444	—	—	—	—	—	0.01
I540	—	0.43	—	0.54	—	0.46	I468	—	0.29	—	0.51	—	0.80
I563	—	0.76	—	0.45	—	0.89	V491	—	0.06	—	0.64	—	0.07
W564	—	0.75	—	0.67	—	0.29	W492	—	0.58	—	1.24	—	1.11
I592	—	0.01	—	0.03	—	0.07	I520	—	0.28	—	—	—	—
Y597	—	1.48	0.01	1.28	0.05	1.66	Y525	—	3.33	—	4.39	—	3.62
Y599	—	2.48	—	1.22	—	1.50	Y527	—	1.65	—	1.83	—	3.24
K601	0.91	0.06	—	0.31	—	0.10	R529	0.88	0.18	0.06	0.07	0.01	1.76
L615	—	0.52	—	0.33	—	0.20	L543	—	0.48	—	0.35	—	0.25
Y642	—	4.38	—	3.04	—	3.94	Y569	—	5.77	—	5.69	—	2.78
T646	—	0.30	—	0.32	—	0.45	T573	—	0.08	—	0.28	—	0.07
S647	0.95	0.31	0.90	0.47	0.97	0.49	E574	0.55	0.07	—	—	—	—
P648	—	1.08	—	0.66	—	0.60	Y575	—	1.59	—	0.17	—	0.36
T649	–—	–	—	0.01	—	0.06	T576	—	—	—	—	—	—
M657	—	0.72	—	1.62	—	0.71	I584	—	0.90	—	0.35	—	0.48
F658	—	—	—	—	—	—	F585	—	—	—	0.05	—	0.01
F681	—	1.09	—	0.24	—	3.42	M608	—	2.20	—	1.28	—	1.34
I682	—	0.16	—	0.34	—	0.16	I609	—	0.40	—	0.66	—	0.39
N705	0.49	1.05	0.44	1.56	0.02	1.04	A632	–	0.26	–	–	–	–
I706	—	0.62	—	0.59	—	0.20	V633	—	0.64	—	0.55	—	0.22
T729	—	0.13	—	0.11	—	—	S655	0.30	0.48	0.16	0.10	0.17	0.02
E730	—	—	—	—	—	—	E656	—	—	—	0.03	—	0.01
ΔH	-40.78 ± 4.36		-35.90 ± 3.53		-37.26 ± 3.68		ΔH	-39.06 ± 3.90		-29.19 ± 3.66		-29.15 ± 4.96	
(kcal/mol)							(kcal/mol)						

## Discussion

Although previous reports strongly suggested that rice BRI1 is a BR receptor in rice [5,9], the binding of BR to rice BRI1 had not been confirmed. In this study, we observed binding between the rice BRI1 core region, which includes the ID and its two neighboring LRRs, with BL (Fig 4). These results suggest that this region is sufficient for BR binding in rice as in Arabidopsis BRI1 [11].

In this study, we investigated two synthetic BRs, iso-carbaBL and 6-deoxoBL, which exhibit different strengths of BR activity in DHE and RLI tests [17]. Feedback regulation of BR biosynthetic gene expression [25,26] is one of the most useful tools to monitor the state of BR signaling. The result of feedback regulation analyses suggests that the regulation is well-correlated with the results of the bioassay (Fig 2). The hypocotyl elongation assay and feedback regulation analysis using *bri1* [19] confirmed that the BR activities of iso-carbaBL and 6-deoxoBL in Arabidopsis are BRI1-dependent (Fig 3). This suggests that the difference between the bioactivities of the two compounds in Arabidopsis and rice is caused before or during the binding of BRs to the receptors. In rice, iso-carbaBL did not show BR activity in the lamina joint or during root elongation. Thus, iso-carbaBL may be inactive in whole rice tissues. We initially hypothesized that iso-carbaBL could bind to Arabidopsis BRI1, but not rice BRI1, and that the different binding abilities of the BRs to BRI1 reflected the BR activity of these chemicals in Arabidopsis and rice. However, the results of the binding assay using BPCS [11] suggest that both iso-carbaBL and 6-deoxoBL can bind to both Arabidopsis and rice BRI1 fragments (Fig 4). Thus, the binding ability of these chemicals to BRI1 is not the cause of their different strengths of BR activity in Arabidopsis and rice.

To explore why iso-carbaBL and 6-deoxoBL exhibited different functions in rice, but not in Arabidopsis, we performed molecular dynamic simulations for a series of complexes. Since no structure of rice BRI1 is available at this time, we generated a modeled rice BRI1 structure based on the Arabidopsis BRI1 –BL complex crystal structure (PDB ID: 3RGZ) [13] using the MODELLER programme. The sequence identity between rice BRI1 and Arabidopsis BRI1 is relatively high (56.3% for T269–D697 of rice BRI1 and S340–D771 of Arabidopsis BRI1). The results of the simulation suggested that rice BRI1 has an ability to bind to BL similar to that of Arabidopsis BRI1, which is consistent with the results of our competitive binding assay (Fig 4). On the other hand, since iso-carbaBL and 6-deoxoBL share a quite similar cholestane structure with BL (Fig 1), BR-binding is not expected to induce large structural changes. The initial BR-complexed structures were built by superimposition of each BR on BL in the BL-Arabidopsis BRI1 complex structure. Collectively, our simulated BRI1 structures may increase our understanding of the binding mechanism of BRs and BRI1s.

The results of the simulation of BL, iso-carbaBL, and 6-deoxoBL against Arabidopsis BRI1 suggested that the binding mechanisms and binding enthalpies were similar, excluding the hydrogen bond between K601 in Arabidopsis BRI1 and the carbonyl groupt at the C6 position of BL because of the lack of oxygen covalently bound to C6 in iso-carbaBL and 6-deoxoBL, which was consistent with the results of our *in vitro* and *in vivo* assays (Figs 1–4). In contrast, in the case of rice BRI1, our simulation suggested that the binding enthalpies with iso-carbaBL and 6-deoxoBL exhibited the large differences from that with BL. Although rice BRI1’s increased binding enthalpy with 6-deoxoBL relative to that with BL, which indicated weaker bindings, was consistent with the results of *in vitro* and *in vivo* assays, the experimental results of the binding between rice BRI1 and iso-carbaBL were inconsistent with the simulated increased binding enthalpy. Although iso-carbaBL did not show BR activity in rice, the results of the binding assays suggested that iso-carbaBL could bind to rice BRI1. Moreover, the angle of the rice lamina joint was increased by BL treatment, but was abolished by co-treatment with iso-carbaBL (Fig 5). These data suggest that iso-carbaBL functions as an antagonist of active BRs in rice. However, why iso-carbaBL functions as an antagonist in rice, but not in Arabidopsis, remains unclear. In addition, why iso-carbaBL shows an antagonistic action, while 6-deoxoBL does not, remains unknown even though both BRs lack the carbonyl group at the C6 position. To address the first question, the molecular dynamics simulation suggested that S647 just before P648 in Arabidopsis BRI1 maintained the position of BRs, as found in the BL-complexed form despite the failure to form the hydrogen bond between K601 and BRs because of the lack of the carbonyl group at the C6 position (Fig 6). However, in rice BRI1, the amino acid at the corresponding position of P648 is tyrosine, Y575, which can change its position more easily than the proline residue. Consequently, rice BRI1 lacked the hydrogen bond between E574 and BRs, which induced the increased binding enthalpy (Fig 6 and Table 1). This may also explain the low BR activity of 6-deoxoBL. For the second question, the simulation suggested that iso-carbaBL inserted more deeply into the rice BRI1 binding pocket upon binding because of the reduced steric restriction caused by the substitution from proline (P648 in Arabidopsis BRI1) to tyrosine (Y575 in rice BRI1) and the addition of the carbonyl group at the carbonyl group at the C6a position, while 6-deoxoBL resisted such deep-insertion because 6-deoxoBL has no modification at the C6a position and may maintain contact with the surface residues, including Y527 (Fig 6 and Table 1). This small difference might be able to explain why iso-carbaBL and 6-deoxoBL function as an antagonist and agonist, respectively. Since our simulation did not consider the binding entropy, especially in the case of iso-carbaBL with rice BRI1, the binding affinity deduced by the only simulated binding enthalpy was not estimated correctly. In fact, the binding of BL to Arabidopsis BRI1 induces the structural change of the loop between the island domain and the LRR core structure [13] where P648 is beside. When iso-carbaBL inserted deeply into rice BRI1, supported by an increased number of contacts between iso-carbaBL and V491, an increased number of water molecules pre-bound to protein may protrude from the BR-binding site, which contributes to the positive binding entropy.

In Arabidopsis, mutations in the ID interfere with binding of the ID (S662F and G611E) or with the hydrogen bonding network (G644D), resulting in the mutant phenotype [12]. Previously, we identified 10 alleles of rice BRI1 [5]. Their phenotypes were not severe, although the amino acid mutations of the rice BRI1 in *d61-7* (A467V), *d61-8* (G522E), and *d61-9* (G539D) were found in or near the core LRR17-ID-LRR18 region. The docking simulation analysis performed in this study suggests that these amino acids do not interact directly with the BL molecule (Fig 6 and Table 1). The *d61-2* (V491M) mutant, which shows a relatively strong phenotype, has an amino acid substitution in the rice BRI1 located in the core region. The simulation analysis suggests that this amino acid residue interacts weakly with BL through a limited number of van der Waals contacts. As mentioned above, iso-carbaBL increased the connection with V491, which was located deeper in the binding pocket. This disordered binding of the compound may block access of active BRs to rice BRI1. In contrast with Arabidopsis BRI1 I563, the calculated interaction of V491 and BL is minimal (Table 1). The change of this amino acid to Met results in the relatively strong BRI1-deficient phenotype (*d61-2*) [5,9], and thus a very strong interaction of BRs with V491 may not be preferable in rice. Thus, this mutation may interfere with the binding of BRs to rice BRI1 and cause the relatively severe phenotype of *d61-2*.

Recently, modified ligand compounds have been synthesised to block the interaction between a receptor and its co-receptor or regulator [27,28]. Brassinolide-2,3-acetonide is one such compound synthesized based on the crystal structure of Arabidopsis BRI1 to antagonise BR signaling [27]. Based on the crystal structure study, this compound may disrupt the interaction of BRI1 with its co-receptor of BRI1, BAK1 [14]. In this study, we hypothesized that iso-carbaBL is an antagonist. The presumed mechanism of the induction of the antagonistic effect of iso-carbaBL is completely different from that of brassinolide-2,3-acetonide; it appears to block access of BL to the BRI1 pocket through deep binding with BRI1. When we compare the functions of the two compounds, the antagonistic effect of iso-carbaBL is relatively weaker than that of brassinolide-2,3-acetonide [27] (Fig 5). The biggest advantage of the effect of iso-carbaBL is species specificity. This characteristic could be used to develop pesticides that function in a species-specific manner.

## Materials and methods

### Chemicals

Iso-carbaBL and 6-deoxoBL were synthesised according to the methods in ref.s [17] and [18].

### Plant materials and growth conditions

Wild-type rice plants (*Oryza sativa* L. cv. Nipponbare) were grown under continuous light at 30°C in a plant box containing MS medium with 3% (w/v) sucrose. Arabidopsis *det2-1* and *bri1-5* mutants were grown in the dark at 22°C in a petri dish containing half-strength MS medium with 1% sucrose.

### Bioassays

Well-grown, uniform seedlings were selected and used for the RLI assay. BRs for the bioassay were dissolved in EtOH and 1 μL was applied inside of the second leaf blade. After incubation for 2 d, the angle of the leaf blade and sheath (A), and the number and size of cells, were measured. The value equal to 180° –A was used as the angle in presented in the results. For each DHE test, approximately 30 seeds were sown on the medium and grown in the dark for 8 d. A total of 15 seedlings were chosen randomly, and their hypocotyl lengths were measured.

### Expression and purification of Arabidopsis and rice BRI1 fragments

Arabidopsis and rice BRI1 subdomains, LRR21-ID-LRR22 (amino acids 556–673) and LRR17-ID-LRR18 (amino acids 484–600), respectively, were amplified by polymerase chain reaction from the full-length BRI1 complementary DNA (clone ID, P0480C01 from Rice Genome Resource Center, http://www.rgrc.dna.affrc.go.jp). The primers used were AtBRI1LRRIDLRR(U): 5’-TTTGAATTCCTCGGCGACTGCAGAAGC-3’ and AtBRI1LRRIDLRR(L): 5’- TTTCTCGAGTCTCCTTCGGTATGTATCCA-3’ for AtBRI1 domains and OsBRI1LRRIDLRR(U): 5’- TTTGAATTCCTCGGTGACTGCCAGAGCTT-3’ and OsBRI1IDLRR(L): 5’- TTTCTCGAGCTCGCCAGGAATCGCCGAGT-3’ for OsBRI1 domains. Amplified fragments were cloned in-frame with glutathione *S*-transferase (GST) between the *Eco*RI and *Xho*I sites of the pGEX6P-1 vector (GE Healthcare). GST fusion proteins were purified with Glutathione-Sepharose 4B beads (GE Healthcare) in accordance with the manufacturer’s instructions.

### Photoaffinity crosslinking of BPCS and detection of binding signal

Photoaffinity labelling was performed according to the method described in ref. [11] with minor modifications. Briefly, we used biotin monoclonal antibody produced in mouse (Amersham Bioscience) or GST polyclonal antibody produced in goat (Jackson Immuno Research Inc.) at a dilution of 1:2000 for primary antibody and rabbit anti-mouse IgG secondary antibody conjugated to horseradish peroxidase (Pierce) for mouse biotin monoclonal antibody or rabbit anti-goat IgG secondary antibody conjugated to horseradish peroxidase for goat GST polyclonal antibody at a dilution of 1:10,000 for immunoblotting. The signal was detected using Immobilon Western Chemiluminescent HRP Substrate (Millipore) and LAS1000 (Fujifilm).

### Quantitative RT-PCR

Arabidopsis (Col-0) and rice (*O*. *sativa* L. cv. Nipponbare) were grown on a medium containing 1/2MS (1% sucrose, 1% agar) for 6 d (Arabidopsis) and 4 d (rice) at 22°C (Arabidopsis) and 30°C (rice) under continuous light and then incubated with liquid 1/2MS medium (1% sucrose) for 1 d following the treatment with BRs for 3 or 6 h. qRT-PCR was performed according to a previous report [29], but we used a Thermal Cycler Dice Real Time System TP900 (Takara) for relative quantification analyses [29]. Primers used to detect gene expression were 5’-GTGATCTCAGCCGTACATTTGGA-3’ and 5’-CACGTCGAAAAACTACCACTTCCT-3’ for *DWARF4*, 5’-CAATAGTCTCAATGGACGCAGAGT-3’ and 5’-AACCGCAGCTATGTTGCATG-3’ for *BR6ox2*, 5’-CCCTCGCCATCTTCTTCCT-3’ and 5’-TGCGTGAAAACCATCTCTTTGT-3’ for *OsDWARF4*, and 5’-TGCTGAGGAAAACTACCCAAGA-3’ and 5’-TCTTCTCCAGCCACCTCCA-3’ for *OsDWARF*.

### Docking simulation analysis

Details of methods and results are provided in the S1 Method and S1 Fig. Briefly, the coordinates of iso-carbaBL and 6-deoxoBL were generated by manual atom-substitution using the coordinates of BL in the complex with Arabidopsis BRI1 (3RGZ) [13]. The model structure of rice BRI1 (from T269 to D697) was generated by MODELLER 9v3 using the same Arabidopsis BRI1 and BL complex structure (from S340 to D771) based on sequential alignment performed using CLUSTAL W software [30]. Each initial complex structure was made by manually fitting the coordinates of the protein and compounds to those of the Arabidopsis BRI1 and BL complex, respectively. Molecular dynamics simulations for a series of complexes were performed using the AMBER9 software package. The binding enthalpy was evaluated using the MM-PBSA procedure in AMBER9. The number of hydrogen bonds and/or van der Waals contacts between protein and compound were calculated using the LIGPLOT programme.

## Supporting information

S1 Fig RMSD curves for Arabidopsis/rice BRI1 Cαs with respect to the initial conformations. (a) Arabidopsis BRI1s docked with BL (left), iso-carbaBL (middle), and 6-deoxoBL (right). (b) Rice BRI1s docked with BL (left), iso-carbaBL (middle), and 6-deoxoBL (right).
(PDF)

S1 Method Docking simulation analysis. (DOCX)
