# Supplementary figures and images for: Molecular actions of two synthetic brassinosteroids, iso-carbaBL and 6-deoxoBL, which cause altered physiological activities between Arabidopsis and rice
Source: PLoS One. 2017 Apr 3;12(4):e0174015. doi: 10.1371/journal.pone.0174015 (PMC5378332; doi:10.1371/journal.pone.0174015)

**a**

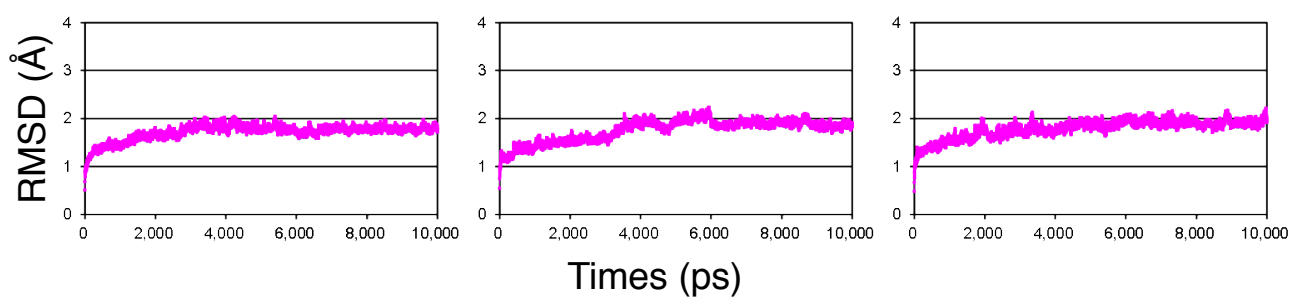

**b**

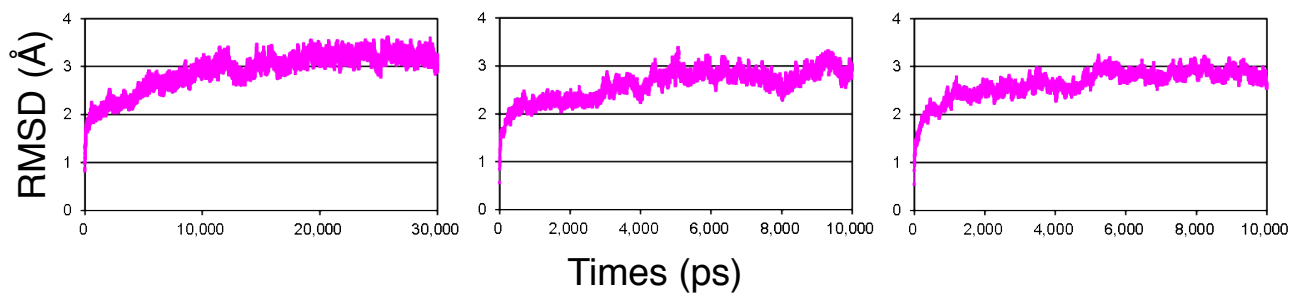

Supplement: S1 Fig — (a) Arabidopsis BRI1s docked with BL (left), iso-carbaBL (middle), and 6-deoxoBL (right). (b) Rice BRI1s docked with BL (left), iso-carbaBL (middle), and 6-deoxoBL (right). (PDF) [file pone.0174015.s001.pdf]
