# Supplementary material for: Molecular actions of two synthetic brassinosteroids, iso-carbaBL and 6-deoxoBL, which cause altered physiological activities between Arabidopsis and rice
Source: PLoS One. 2017 Apr 3;12(4):e0174015. doi: 10.1371/journal.pone.0174015 (PMC5378332; doi:10.1371/journal.pone.0174015)
Supplement: S1 Method — (DOCX) [file pone.0174015.s002.docx]

**Supplementary Methods**

**Docking simulation analysis**

To evaluate the binding energies of Arabidopsis and rice BRI1 against BL, iso-carbaBL, and 6-deoxoBL, we used a PDB file containing the structure of the Arabidopsis BRI1 and the docked BL compound (PDBID: 3RGZ [1]) as a template. The 3D model of the rice BRI1 was built based on Arabidopsis BRI1 structure using MODELLER program [2]. The hetero-atom module in the MODELLER was used to model the rice BRI1 docked with BL, which assumes that the ligand interacts similarly as shown in the Arabidopsis BRI1 and BL complex because the amino acid types of the residues involved in the interaction site estimated by LIGPLOT [3] for the 3RGZ coordinate are almost the same as those of the corresponding residues in rice BRI1 except Ser647 and Pro648 in Arabidopsis BRI1, while Glu574 and Tyr575 in rice BRI1. ANTECHAMBER module [4] implemented in the AMBER [5] package was used to automatically assign the parameters to BL, iso-carbaBL, and 6-deoxoBL using general AMBER force field and create each topology file. This module requires only a three-dimensional structure as input. The coordinates of iso-carbaBL and 6-deoxoBL were generated manually based on that of BL in 3RGZ. Each complex structure was created by merging the coordinate of each docked ligand with each protein, where the relative positon of ligand molecule to protein was manually set as in 3RGZ. After 500-step minimization process in vacuum, the all systems were solvated with explicit TIP3P water molecules under periodic boundary conditions using AMBER software [5]. The initial systems were minimized and subsequently heated from 50 K to 300 K with 200 ps simulation time. Molecular dynamics simulations were then continued in the NPT ensemble at 300 K and 1 bar pressure. The all systems except the rice BRI1 docked with BL system attained equilibrium within 10 ns-simulation after some initial fluctuations, while the rice BRI1 docked with BL system did not reached equilibrium within 10 ns-simulation (Supplementary Figures). Thus, the simulation for the rice BRI1−BL system was continued up to 30 ns. For the energetic analysis, we extracted each 200-snapshot from the final 1 ns-trajectory for each system (for rice BRI1−BL system, 600-snapshots from the final 3 ns-trajectory). MM-PBSA script implemented in AMBER software were used for free energy calculation. We did not perform the calculations of the entropy contributions to binding, therefore, our results were not true free energy values but could be used to compare among similar systems.

**Supplementary References**

1. She J. Han Z, Kim T-W, Wang J, Cheng W, Chang J, et al. Structural insight into brassinosteroid perception by BRI1. Nature 2011;474: 472-476.

2. Eswar N, Webb B, Marti-Renom MA, Madhusudhan M, Eramian D, Shen M-y, et al. in Current Protocols in Protein Science; 2007 Chapter 2, Unit 2.9.

3. Wallance AC, Laskowski RA, Thornton JM LIGPLOT: a program to generate schematic diagrams of protein-ligand interactions. Protein Eng*.* 1996;8: 127-134.

4. Wang J, Wang W, Kollman PA, Case DA Automatic atom type and bond type perception in molecular mechanical calculations. J. Mol. Graph. Model. 2006;25: 247–260.

5. Case DA, Cheatham TE, Darden T, Gohlke H, Luo R, Merz KM, et al. The Amber biomolecular simulation programs. J Comput Chem*.* 2005;26: 1668–1688.
